# Annual decline rate in FEV1s in community-dwelling older adults diagnosed with mild to moderate COPD

**DOI:** 10.1038/s41533-022-00292-w

**Published:** 2022-08-26

**Authors:** Aldana Rosso, Karl Egervall, Sölve Elmståhl

**Affiliations:** Division of Geriatric Medicine, Department of Clinical Sciences in Malmö, Lund University, Malmö, Sweden

**Keywords:** Chronic obstructive pulmonary disease, Epidemiology

## Abstract

Information about the decline rate in forced expiratory volume in 1 s (FEV1s) in older adults with COPD is scarce. A total of 4082 community-dwelling older adults from the population-based study Good Aging in Skåne were followed for 12 years and 143 participants developed COPD. The average FEV1s decline is estimated to be 66.3 mL/year, (95% CI [56.4; 76.3]) and 43.3 mL/year (1.7%/year, 95% CI [41.2; 45.5]) for COPD and non-COPD participants, respectively.

The forced expiratory volume in 1 s (FEV1s) decline rate in people aged ≥ 65 years ranges from 17.7 to 46.4 mL/year^1^. The decline rate in FEV1s due to chronic obstructive pulmonary disease (COPD) is accelerated compared to natural decline. In adults aged 48–64 years, conflicting results for the FEV1s annual decline rate in mild to moderate COPD patients have been reported (33–129 mL/year^2–6^). Studies regarding the lung function decline in early-stage COPD patients diagnosed above 65 years of age are scarce^1,7^. We aim to estimate the FEV1s annual decline rate in community-dwelling older adults diagnosed with early-stage COPD and to investigate whether it differs from that due to normal aging.

Baseline characteristics are shown in Table 1. One-hundred and forty-three of the 4082 included participants were diagnosed with COPD in standard clinical practice after the baseline visit. The COPD prevalence at the first visit was 5.5% (95% CI [5.0–6.1]). The incidence rate of COPD was 10.6 cases per 1000 person/years (95% CI [9.0; 12.4]).

**Table 1:** Baseline characteristics.

Baseline characteristics		Developed COPD during follow-up (*n* = 143)	Did not develop COPD during follow-up (*n* = 3939)	All participants (*n* = 4082)
Sex (*n*, %)	Male	63 (44.1)	1867 (47.4)	1930 (47.3)
	Female	80 (55.9)	2072 (52.6)	2152 (52.7)
Smoking status (*n*, %)	Current smoker	65 (45.4)	487 (12.4)	552 (13.5)
	Former smoker	53 (37.1)	1614 (41.0)	1667 (40.8)
	Never smoker	25 (17.5)	1778 (45.1)	1803 (44.2)
	Missing	0 (0)	60 (1.5)	60 (1.5)
Heart disease including hypertension (*n*, %)	No	4 (2.8)	228 (5.8)	232 (5.7)
	Yes	139 (97.2)	3711 (94.2)	3850 (94.3)
Cerebrovascular disease (*n*, %)	No	125 (87.4)	3502 (88.9)	3627 (88.8)
	Yes	18 (12.6)	437 (11.1)	455 (11.2)
Diabetes type I and II (*n*, %)	No	135 (94.4)	3588 (91.1)	3723 (91.2)
	Yes	8 (5.6)	351 (8.9)	359 (8.8)
Asthma (*n*, %)	No	108 (75.5)	3667 (93.1)	3775 (92.5)
	Yes	35 (24.5)	272 (6.9)	307 (7.5)
Age (years)	mean (std) [min, max]	69.1 (9.37) [59.58,93.06]	70.44 (10.64) [59.18,94.94]	70.39 (10.6) [59.18,94.94]
Body mass index (kg/m2)	mean (std) [min, max]	26.79 (4.59) [18.14,48.7]	27.23 (4.35) [16.32,54.14]	27.22 (4.36) [16.32,54.14]
	Missing	0 (0)	36 (0.9)	36 (0.9)
Education (years)	mean (std) [min, max]	10.0 (3.5) [6.0,23.0]	10.63 (3.71) [1.0,30.0]	10.61 (3.7) [1.0,30.0]
	Missing		32 (0.8)	32 (0.8)
Forced Expiratory Volume in 1 s (L)	mean (std) [min, max]	2.09 (0.7) [0.76,3.86]	2.56 (0.85) [0.38,5.74]	2.54 (0.85) [0.38,5.74]
Forced Expiratory Volume in 1 s/forced volume capacity	mean (std) [min, max]	0.69 (0.11) [0.39,0.97]	0.78 (0.08) [0.34,1.0]	0.78 (0.08) [0.34,1.0]
Forced Expiratory Volume in 1 s/forced volume capacity below lower limit of normal	No	97 (67.8)	3756 (95.4)	3853 (94.4)
	Yes	46 (32.2)	153 (3.9)	199 (4.9)
	Missing	0 (0)	30 (0.7)	30 (0.7)
Follow-up time (years)	mean (std) [min, max]	7.4 (2.9) [2.0-12]	3.3 (3.8) [0-12]	3.4 (3.9) [0-12]
Attended visits during follow-up	mean (std) [min, max]	2.5 (0.6) [2–4]	1.7 (0.8) [1–5]	1.7 (0.8) [1–5]
Age at COPD diagnosis (years)		74.1 (8.9) [60.9-94.5]	-	-
COPD category 0 (*n*, %)		42 (29.3)	-	-
COPD category 1 (*n*, %)		26 (18.2)	-	-
COPD category 2 (*n*, %)		55 (38.5)	-	-
COPD category 3 (*n*, %)		19 (13.3)	-	-
COPD category 4 (*n*, %)		1 (0.7)	-	-
Treatment for COPD including long-acting β2-agonists, long-acting muscarinic antagonists, corticosteroids, and/or combination therapy (*n*, %)		32 (22.3)	108 (2.7)	140 (3.4)

The average FEV1s decline rate is estimated to be 66.3 mL/year (2.8%/year, 95% CI [56.4; 76.3], *p*-value < 0.001) and 43.3 mL/year (1.7%/year, 95% CI [41.2; 45.5], *p*-value < 0.001) for the COPD and non-COPD participants, respectively. Participants diagnosed with COPD have an additional decline of 22.3 mL/year (1.0%/year, 95% CI [12.8; 33.3], *p*-value < 0.001) compared to non-COPD peers (Supplementary Tables 2, 3, and 4). The estimates are consistent with those obtained in the sensitivity analyses (see Supplementary Table 5). No difference was observed in the mean FEV1s for participants diagnosed before/after 70 years of age (57.2 and 50.0 mL/year, respectively).

The estimated prevalence and incidence of COPD are concordant with previously reported values^8,9^. As also seen in younger COPD patients^2,4,5,10^, a large variability in FEV1s was observed. The estimated mean annual FEV1s decline in COPD (66.3 mL/year, 2.8%/year) is in the upper range of previously reported values for younger COPD patients (33–129 ml/year^2–6^) and larger than the decline rates for older adults reported previously^11^ (49.1 mL/year and 38.3 mL/year for males and females, respectively). The observed average difference in mean annual decline rate between early-stage COPD and non-COPD participants is −23.0 mL/year. In other words, the contribution of early-stage COPD to the FEV1s decline in older adults is about—1.0%/year.

Lung function is inversely associated with heart and cerebrovascular diseases, neurological deficits, diabetes, and metabolic syndrome. On average, 50% of adults aged ≥ 70 years have at least three chronic diseases^12^. Thus, the observed decline rate in FEV1s is explained by a combination of loss of lung function due to aging (e.g., worsening of lung elasticity, weakened muscles, decrease surface area for alveolar gas exchange) and co-existing comorbidities.

The interpretation of our results is affected by the study design and the difficulties of establishing an accurate COPD diagnosis in older adults. Healthy participants are over-represented in the study. Patients were diagnosed according to standard clinical routines and there is risk of COPD misclassification^13^. Interestingly, participants who developed COPD during follow-up, had on average a lower FEV 1s at the first study visit, which could be an indication of underdiagnosis of heart disease, COPD, and/or asthma. Some diagnosed COPD participants had study spirometries within the normal range. Given the large variability observed in spirometries and the fact that most of COPD diagnosed participants are at an early stage of the disease, it is not possible to distinguish misclassified COPD cases from correct diagnosed COPD participants who had a normal spirometry during the study visit. Subjects with comorbidity and inconclusive spirometry results may not have been properly diagnosed in clinical practice. The consequence of this possible misclassification would most likely result in an underestimation of true decline in early onset COPD. Nevertheless, our estimates seem to be robust towards a potential COPD misclassification since concordant results were obtained in a sensitivity analysis where the diagnosis was based solely on study spirometry. We also acknowledge the existence of several COPD phenotypes which might express different trajectories. It was beyond our reach to estimate different decline rates for different COPD phenotypes.

In summary, this study indicates that the FEV1s annual decline rate in early-stage COPD older adults is slightly higher than the decline rate observed in peers without COPD.

## Methods

### Study population

**Fig. 1:**
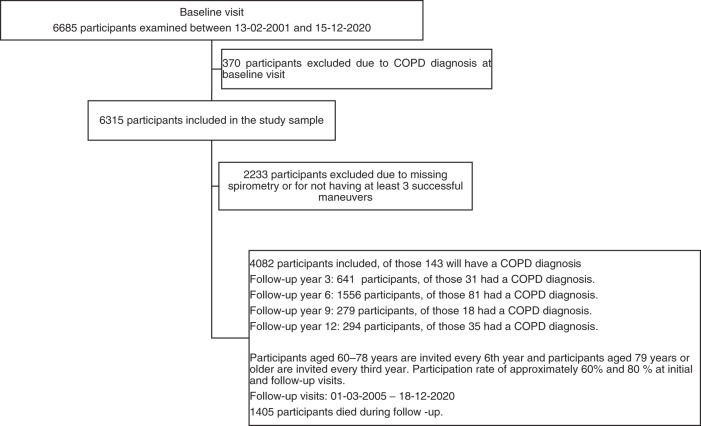
Study population. Participants diagnosed with COPD, with missing spirometry or unable to perform three successful manoeuvres, at baseline were excluded from this study. During follow-up 143 participants received a COPD diagnosis in clinical practice.

As shown in Fig. 1, data were retrieved from the on-going population-based study Good Aging in Skåne (GÅS)^14^. Briefly, 60- to 93-year-old subjects living in Skåne, Sweden, are randomly invited using the population register. Participants are offered a thorough physical, medical, and psychological examination and are invited to follow-up examinations at regular intervals until death. To encourage participation of frail adults, the study team performs home visits following the same study protocol. Three waves have been fully recruited with an initial participation rate of approximately 60%. Participants recruited during the first wave in 2001–2004 were 60, 66, 72, 78, 81, 84, 87, 90, or 93 years old. Participants recruited during the second wave in 2006–2013 were 60, 66, or 81 years old. Participants recruited during third wave in 2012–2016 were 60 or 81 years old. A fourth wave is being recruited since 2019 including participants who are 60 or 81 years old. The GÅS study is conducted according to the Declaration of Helsinki and Good Clinical Practice guidelines and is approved by the Lund University Ethics Review Board (LU 744–00). All participants provide written informed consent. In this study, we aim to study early onset progression, and thus to minimize bias participants with a COPD diagnosis at baseline were excluded from the analysis.

### Spirometry assessments

Spirometry assessments were performed using a Vitalograph 2120 spirometer (Vitalograph Ltd, Buckingham, UK) according to the American Thoracic Society guidelines^15^. Bronchodilators were not administrated during the first wave baseline visit. Subjects received 1.0 mg of β2-receptor agonist terbutaline 10 min prior to the spirometry at all other visits.

### Identification of comorbidities and definition of COPD

Comorbidities were identified during the medical examination, and by retrieving medical records and diagnosis codes from the Skåne Healthcare Registry^16^ (see Supplementary Table 1). Information about prescribed medicines was obtained from medical records and self-reported. The COPD diagnosis was retrieved from medical records. Clinically, diagnosis of COPD in the region of Skåne is based on three criteria: spirometry verified obstructivity (FEV1/FVC < 0.7 after bronchodilation), current airway symptoms, and a history of a risk factor for COPD. COPD diagnosis also included emphysema and chronic bronchitis.

COPD was graded according to the Global Strategy for the Diagnosis, Management, and Prevention of Chronic Obstructive Lung Disease (GOLD) classification^17^ using the spirometries collected during the study visits. In some cases, participants diagnosed with COPD had a normal spirometry during the study visit. Therefore, an additional category level (category 0) was included to accommodate diagnosed subjects whose FEV1s/FVC ratio was ≥ 0.7^5^. The spirometry performed at the study visit was used for the COPD GOLD classification. The reference equations for lower limit of normal are only available for subjects younger than 95 years old and for this calculation, age was truncated at 95 years.

### Statistical analysis

The aim of the statistical analysis was to estimate the average annual decline rate in FEV1s in COPD patients at early COPD onset and to compare it to the decline rate in participants without COPD. The annual change in FEV1s was defined as the difference in FEV1s between two consecutive study visits, divided by the time passed between the visits. This model assumes that the annual decline rate is constant over time. A mixed model for repeated measures with random intercept (participants) was implemented. The variables FEV1s, age, sex, smoking status, education, body mass index, heart disease, cerebrovascular disease, asthma, and diabetes (at baseline) were included to mitigate confounding.

Estimating the difference in annual decline rate between participants with and without a COPD diagnosis is challenging since the two groups should only differ in their COPD status. Patients in clinical practice do not perform spirometry examinations in a regular way. Therefore, it could be more likely for a participant with a low FEV1s to get a spirometry examination and thus a COPD diagnosis compared to a participant with higher FEV1s values regardless of their true COPD status. The previous value of FEV1s may affect the likelihood of getting a COPD diagnosis and thus time-varying confounding may be present. In order to minimize bias, sensitivity analyses using a marginal structural mixed model were performed (see Supplementary Table 5). The risk for misclassification in the COPD diagnosis is acknowledged. We, therefore, presented a Supplementary Note about sensitivity analyses where the COPD status was assigned using the spirometry results obtained at the study visits. The statistical software Stata IC 14.2 (StataCorp LLC, Texas, USA) was used.

### Reporting summary

Further information on research design is available in the Nature Research Reporting Summary linked to this article.

## Supplementary information

Supplementary Material

Reporting Summary

## Data Availability

Data are accessible on request (https://neardb.near-aging.se/study/gas-snac-s).
