# Supplementary material for: Annual decline rate in FEV1s in community-dwelling older adults diagnosed with mild to moderate COPD
Source: NPJ Prim Care Respir Med. 2022 Aug 26;32:30. doi: 10.1038/s41533-022-00292-w (PMC9418326; doi:10.1038/s41533-022-00292-w)
Supplement: Supplementary file 1 — Supplementary Material [file 41533_2022_292_MOESM1_ESM.docx]

**Annual decline rate in forced expiratory volume in 1 secondFEV1s in community-dwelling older adults diagnosed with mild to moderate COPD**

Aldana Rosso^1^, Karl Egervall^1^, Sölve Elmståhl^1^

^1^Division of Geriatric Medicine, Department of Clinical Sciences in Malmö, Lund University, Malmö, Sweden.

**SUPPLEMENTARY MATERIAL**

**SUPPLEMENTARY METHODS**

**Disease codes classification according to the Classification of Diseases system version 10 (ICD-10)**

| Heart disease | I 20 | Angina pectoris |
| --- | --- | --- |
|  | I 21 | Acute myocardial infarction |
|  | I22 | Subsequent ST elevation (STEMI) and non-ST elevation (NSTEMI) myocardial infarction |
|  | I23 | Certain current complications following ST elevation (STEMI) and non-ST elevation (NSTEMI) myocardial infarction (within the 28-day period) |
|  | I24 | Other acute ischemic heart diseases |
|  | I25 | Chronic ischemic heart disease |
|  | I50 | Heart failure |
|  | I48 & I49 | Atrial fibrillation |
|  | Z95 | Presence of cardiac and vascular implants and grafts |
| Cerebrovascular disease | I60 | Nontraumatic subarachnoid haemorrhage |
|  | I61 | Nontraumatic intracerebral haemorrhage |
|  | I62 | Other and unspecified nontraumatic intracranial haemorrhage |
|  | I63 | Cerebral infarction |
|  | I64 | Stroke, not specified as haemorrhage or infarction |
|  | I65 | Occlusion and stenosis of precerebral arteries, not resulting in cerebral infarction |
|  | I66 | Occlusion and stenosis of cerebral arteries, not resulting in cerebral infarction |
|  | I67 | Other cerebrovascular diseases |
|  | I68 | Cerebrovascular disorders in diseases classified elsewhere |
|  | I69 | Sequelae of cerebrovascular disease |
|  | G45.x | Transient cerebral ischemia (TIA) |
|  |  |  |
| Dementia | F00 | Dementia in Alzheimer disease |
|  | F01 | Vascular dementia |
|  | F02 | Dementia in other diseases classified elsewhere |
|  | F03 | Unspecified dementia |
|  |  |  |
| Asthma | J45 | Allergic (predominantly) asthma, allergic bronchitis NOS, allergic rhinitis with asthma, atopic asthma, extrinsic allergic asthma, hay fever with asthma, idiosyncratic asthma, intrinsic nonallergic asthma, nonallergic asthma |
| Hypertension | I10 | Essential (primary) hypertension |
|  | I 11 | Hypertensive heart disease |
|  | I 12 | Hypertensive renal disease |
|  | I13 | Hypertensive heart and renal disease |
|  | I15 | Secondary hypertension |
|  |  |  |
| Diabetes | E10 | Type 1 diabetes mellitus |
|  | E11 | Type 2 diabetes mellitus |
|  | E12 | Malnutrition-related diabetes mellitus |
|  | E13 | Other specified diabetes mellitus |
|  | E14 | Unspecified diabetes |
|  |  |  |
| Hypercholesterolemia | E78.0 | Pure hypercholesterolaemia |
|  |  |  |
| COPD | J44 | asthma with chronic obstructive pulmonary disease, chronic asthmatic (obstructive) bronchitis, chronic bronchitis with airways obstruction, chronic bronchitis with emphysema, chronic emphysematous bronchitis, chronic obstructive asthma, chronic obstructive bronchitis |
|  | J41 | Simple and mucopurulent chronic bronchitis |
|  | J43 | Emphysema |
|  | J44.1 | Exacerbations |

Supplementary Table 1 Diagnosis codes

**Sample size calculation**

The number of participants attending the GÅS study was decided several years before the initiation of this study. Therefore, it was not possible to influence the sample size. In addition, the wide range of reported estimates of decline in FEV1s in adults makes it difficult to target a specific effect size. Notwithstanding the mentioned constrains, the number of COPD participants in the database was roughly estimated before initiating this study to assess the feasibility of running a mixed model. Assuming that the COPD prevalence in GÅS was similar to that in the overall population (about 7 %), the attendance rate was 60 %, and that 40 % of participants would not have a complete spirometry assessment, over 100 incident COPD cases were expected. This was considered to be sufficient to achieve convergence of the mixed model and thus the study sample was requested from the GÅS committee.

**SUPPLEMENTARY NOTES**

**Results for the primary analyses**

The primary analysis consisted of the estimation of a linear mixed model for the change per year in FEV1s for participants with and without a COPD diagnosis. In order to mitigate bias, the mixed model included baseline characteristics as covariates. The results for the model coefficients are shown below. Since our model is optimized for the interpretation of the coefficient related to COPD, and the other model coefficient were included only for confounding purposes, no conclusion can be drawn regarding the effect of the other covariates on the FEV1s decline rate^1^.

| **Parameter** | **Coefficient** | **p-value** | **Lower 95 % CI** | **Upper 95 % CI** |
| --- | --- | --- | --- | --- |
| Sex (ref. male) | -0.008 | 0.007 | -0.015 | -0.002 |
| FEV1s at first visit (L) | -0.025 | 0.000 | -0.030 | -0.020 |
| BMI at first visit (Kg/m2) | 0.001 | 0.026 | 0.000 | 0.001 |
| Age at first visit (year) | -0.001 | 0.000 | -0.001 | -0.001 |
| Smoking status at first visit (ref never smoker) |  |  |  |  |
| Previous smoker | 0.004 | 0.145 | -0.001 | 0.008 |
| Current smoker | -0.008 | 0.006 | -0.015 | -0.002 |
| Heart disease present at baseline (Ref No) | -0.002 | 0.821 | -0.015 | 0.012 |
| Cerebrovascular disease present at baseline (Ref No) | -0.0002 | 0.970 | -0.011 | 0.011 |
| Diabetes present at baseline (Ref No) | -0.004 | 0.365 | -0.012 | 0.004 |
| Asthma present at baseline (Ref No) | -0.001 | 0.712 | -0.009 | 0.006 |
| COPD (ref No) | -0.023 | 0.000 | -0.033 | -0.013 |
| Intercept | 0.0925 | 0.000 | 0.0541 | 0.1301 |

Supplementary Table 2 Model parameters

**Estimates for decline rate in FEV1s according to the smoking status**

|  | **Estimated mean annual rate FEV1s (mL/Year)** | **Lower 95 % CI** | **Upper 95 % CI** |
| --- | --- | --- | --- |
| Never smoker, no COPD | -43.7 | -47.0 | -40.7 |
| Never smoker, COPD | -66.7 | -77.2 | -56.2 |
| Former smoker, no COPD | -40.1 | -43.7 | -36.5 |
| Former smoker, COPD | -63.1 | -73.3 | -52.9 |
| Current smoker, no COPD | -52.2 | -57.6 | -46.7 |
| Current smoker, COPD | -75.2 | -85.4 | -65.0 |

Supplementary Table 3 Estimated decline rate in FEV1s according to the smoking status

**Estimates for decline rate in FEV1s for subjects with and without asthma diagnosis at baseline**

|  | **Estimated mean annual rate FEV1s (mL/Year)** | **Lower 95 % CI** | **Upper 95 % CI** |
| --- | --- | --- | --- |
| No asthma at baseline, no COPD | -43.2 | -45.5 | -41.0 |
| No asthma at baseline, COPD | -66.2 | -76.2 | -56.2 |
| Asthma at baseline, no COPD | -44.7 | -52.1 | -37.2 |
| Asthma at baseline, COPD | -67.6 | -79.3 | -56.0 |

Supplementary Table 4 Estimated decline rate in FEV1s for subjects with and without asthma diagnosis at baseline

**Sensitivity analyses to investigate the risk of time-varying confounding**

Estimating the difference in decline rate between participants with and without a COPD diagnosis is challenging since we need to make the two groups somewhat comparable, so they only differ in their COPD status. Patients in clinical practice do not perform spirometry examinations in a regular way. Therefore, it could be more likely for a participant with a low FEV1s to get a spirometry examination and thus a COPD diagnosis compared to a participant with higher FEV1s values regardless of their true COPD status. The previous value of FEV1s may affect the likelihood of getting a COPD diagnosis. In other words, there is feedback between the exposure (COPD diagnosis) and the outcome (FEV1s). Unfortunately, time-varying confounding cannot be corrected using “traditional” adjustments and other statistical methods are required**^2,3^**. We performed sensitivity analyses using a marginal structural mixed model to adjust for potential time-varying confounding. Inverse probability weighting (IPW) was used to create a pseudo-population in which FEV1s is independent of COPD diagnosis. In this way, the COPD participants are comparable to non-COPD participants at each time point. Logistic regression was implemented to separately estimate the probability of developing COPD at each visit. Baseline characteristics and the FEV1s value observed at the previous visit were used as covariates to estimate the probability of COPD at each follow-up. Regular, standardized, and truncated (5^th^ percentile) weights were tested. Inverse probability weighting can only handle a monotonous missing pattern, and thus participants with intermittent missing pattern were excluded. To avoid extreme weights, only data from up to 4 follow-up visits per participant were used in the sensitivity analyses. For these models only a random intercept for participants was considered. Confidence intervals were calculated using robust standard errors.

It is worth noticing that marginal structural models adequately control bias due to time-varying confounding under the strict assumptions of positivity and unmeasured confounding. Positivity can, to a certain extent, be investigated by assuring that the probability of attendance and COPD diagnosis are different from zero for all subjects. Despite collecting extensive information about health status and lifestyle, unmeasured confounding remains. Therefore, the results of these analyses are only considered supportive.

| **Model** | **Estimated mean annual rate FEV1s (mL/Year) for COPD participants (95 % CI)** | **Estimated annual rate FEV1s (mL/Year) for non-COPD participants (95 % CI)** |
| --- | --- | --- |
| Standard weights | -67.4 (-80.7; - 54.1) | -42.3 (-44.7; - 39.8) |
| Truncated standard weights at 5th percentile | -69.0 (-81.1; - 57.0) | -42.1 (-44.5; - 39.7) |
| Stabilized weights | -66.8 (-79.1; - 54.4) | -41.3 (-43.8; - 38.9) |

Supplementary Table 5 Sensitivity analyses using a marginal structural mixed model to adjust for potential time varying confounding

**Sensitivity analyses to investigate the risk of COPD misclassification**

In the primary analysis, we assumed that the COPD diagnosis assigned by the treating physician was correct. Clinically, the diagnosis of COPD in the region of Skåne is based on three criteria: spirometry verified obstructivity (FEV1/FVC < 0.7 after bronchodilation), current airway symptoms, and a history of a risk factor for COPD. Nevertheless, we recognize that a certain misclassification in the COPD diagnosis may have occurred. Therefore, we performed a sensitivity analysis where the COPD diagnosis was assigned solely using the study spirometry. Study participants with FEV / FVC < 0.7 were classified as participants with COPD. We included data from 4,330 participants who performed at least 3 correct spirometry manoeuvres. Of those, 731 participants were excluded from the analysis since they have a FEV / FVC < 0.7 at baseline. Finally, records from 3,599 subjects were included. Of those, 307 developed “COPD” according to the new definition (FEV /FVC < 0.7). The same mixed model implemented in the primary analysis was used. The estimated average decline rate in FEV1s was 68.1 mL/year 95 % CI (59.6; 76.6) and 40.9 mL/year 95 % CI (38.7; 43.1) and for subjects with COPD and without COPD, respectively. These estimates are very similar to those reported in the primary analysis (66.3 mL/year and 43.3 mL/year).

**SUPPLEMENTARY REFERENCES**

1 Westreich, D. & Greenland, S. The Table 2 Fallacy: Presenting and Interpreting Confounder and Modifier Coefficients. *American Journal of Epidemiology* **177**, 292-298, doi:10.1093/aje/kws412 (2013).

2 Cole, S. R. & Hernan, M. A. Constructing inverse probability weights for marginal structural models. *Am J Epidemiol* **168**, 656-664, doi:10.1093/aje/kwn164 (2008).

3 Mansournia, M. A., Etminan, M., Danaei, G., Kaufman, J. S. & Collins, G. Handling time varying confounding in observational research. *BMJ* **359**, j4587, doi:10.1136/bmj.j4587 (2017).
